# Roth Spots: A Rare Finding in Sickle Cell Anemia

**DOI:** 10.7759/cureus.59047

**Published:** 2024-04-26

**Authors:** Manjeet Kothari, Anjalee Chiwhane, Sunil Kumar, Anil Wanjari, Harshitha Reddy

**Affiliations:** 1 Internal Medicine, Jawaharlal Nehru Medical College, Wardha, IND

**Keywords:** endocarditis, hemorrhage, sickle cell anemia, hbss, roth spot

## Abstract

White-centered, flame-shaped retinal hemorrhages, also known as Roth spots, occur in several diseases, most often in subacute bacterial endocarditis. Other conditions such as leukemia, hypertensive retinopathy, and pre-eclampsia are other causes. Here, we present the case of a 32-year-old female with sickle cell anemia (HbSS) who was treated at the hospital after experiencing a sickle cell crisis. Fundus examination was performed to rule out sickle cell retinopathies, suggesting the presence of Roth spots in the retina which are rarely seen in sickle cell disease.

## Introduction

Sickle cell is an inherited hemoglobin disorder caused by homozygosity of the βS type of allele on the 11p15.5 chromosome. This genetic condition leads to substituting a glutamic acid, which is hydrophilic, with valine, which is hydrophobic at the sixth location in the β-chain. As a result, individuals with this disease have a mutated tetramer of hemoglobin known as HbS (α2βs2) in their erythroid cells. It poses unique challenges due to its impact on the vascular system [1].

Roth spots, characterized by white-centered retinal hemorrhages, serve as important clinical indicators associated with a spectrum of medical conditions. While originally linked to subacute bacterial endocarditis, their presence has been documented in many conditions, including leukemia, hypertensive retinopathy, diabetic retinopathy, anemia, and pre-eclampsia. Among the myriad conditions associated with Roth spots, sickle cell anemia is a relatively uncommon but noteworthy entity [2]. Various theories attempt to explain the origin of retinal hemorrhages with white centers, with the prevailing view attributing them to retinal capillary rupture due to endothelial cell dysfunction. Following vessel rupture, red blood cells leak out, which triggers the clotting chain reaction and forms fibrin-platelet plugs on the damaged endothelial cell lining. Histological studies confirm that the white lesions contain platelet-fibrin thrombi [3].

## Case presentation

A 32-year-old female with a history of homozygous (HbSS) sickle cell disease came to the emergency of the hospital with complaints of fever, chest pain, knee pain, and abdominal pain for three days. She had a history of multiple admissions with blood transfusions for similar complaints in the past.

On physical examination, the patient’s blood pressure was 100/80 mmHg, pulse rate was 122 beats/minute, respiratory rate was 28 breaths/minute, and SpO_2_ was 96% while breathing ambient room air. The patient was febrile to touch. On further examination, the patient had pallor and icterus. The remainder of the physical examination was otherwise unremarkable. Table 1 displays the results of the patient’s laboratory tests.

**Table 1:** Patient’s laboratory findings.

Laboratory investigation	Value	Biological laboratory reference
Hemoglobin	5.6	13–15 g/dL
Total leucocyte count	4,600	4,000–11,000/mm^3^
Platelet	350,000	150,000–450,000/mm^3^
Mean corpuscular volume	87.8	79–100 fL
Hematocrit	28.8	35–40%
Reticulocyte count	0.3	0.5–2.5%
Urea	16	9–20 mg/dL
Creatinine	1	0.6–1.2 mg/dL
Sodium	139	137–145 mmol/L
Potassium	3.8	3.5–5.1 mmol/L
Alkaline phosphatase	80	38–126 U/L
Alanine transaminase	52	<50 µ/L
Aspartate transaminase	45	17–59 µ/L
Total protein	6.2	6.3–8.2 g/dL
Albumin	3.1	3.5–5 g/dL
Total bilirubin	1.8	0.2–1.3 mg/dL
Conjugated bilirubin	0.6	0.0–0.3 mg/dL
Unconjugated bilirubin	1.2	0.0–1.1 mg/dL
Globulin	3.1	2.3–3.5 g/dL
Ferritin	270	6.24–137 ng/mL

**Figure 1:**
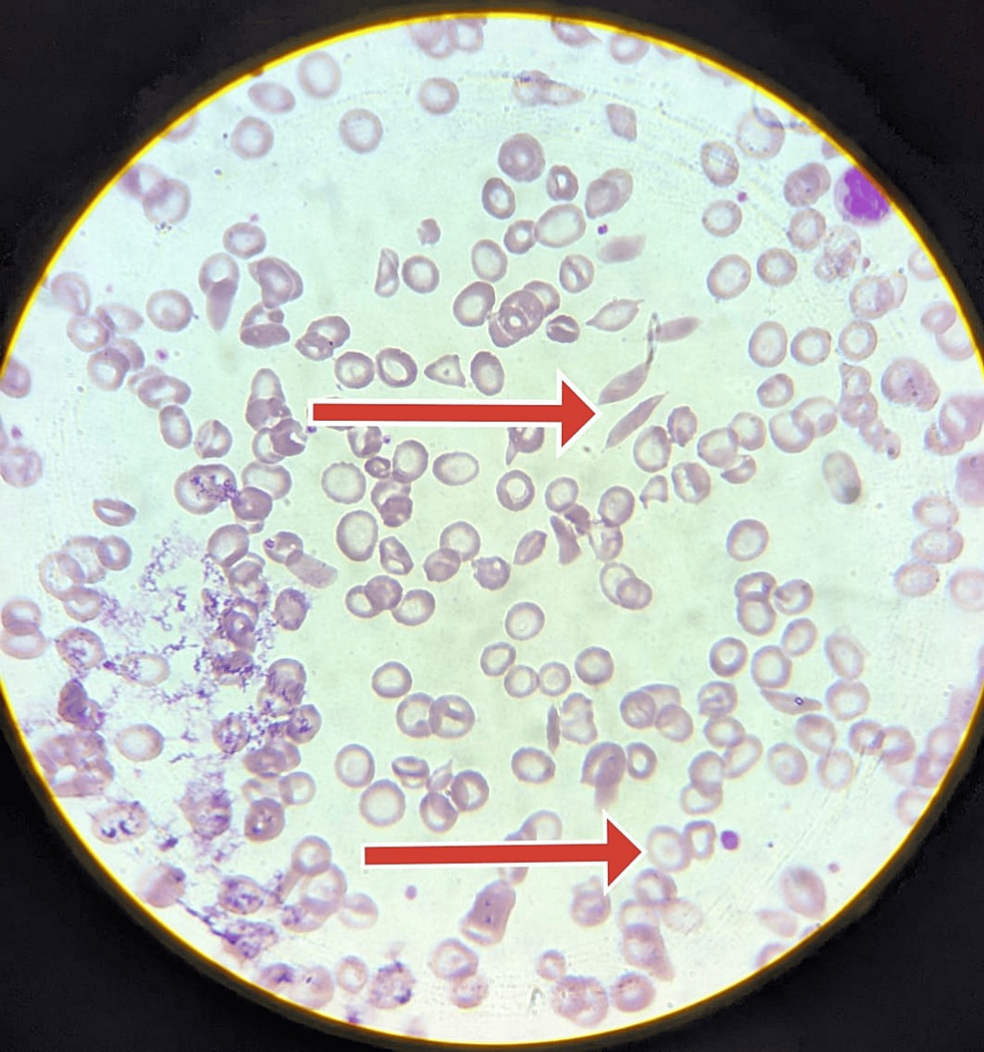
High-power view of peripheral smear stained with Leishman stain shows normocytic normochromic red blood cells. Peripheral smear also shows tactoids (red arrow) and occasional target cells (red arrow).

**Figure 2:**
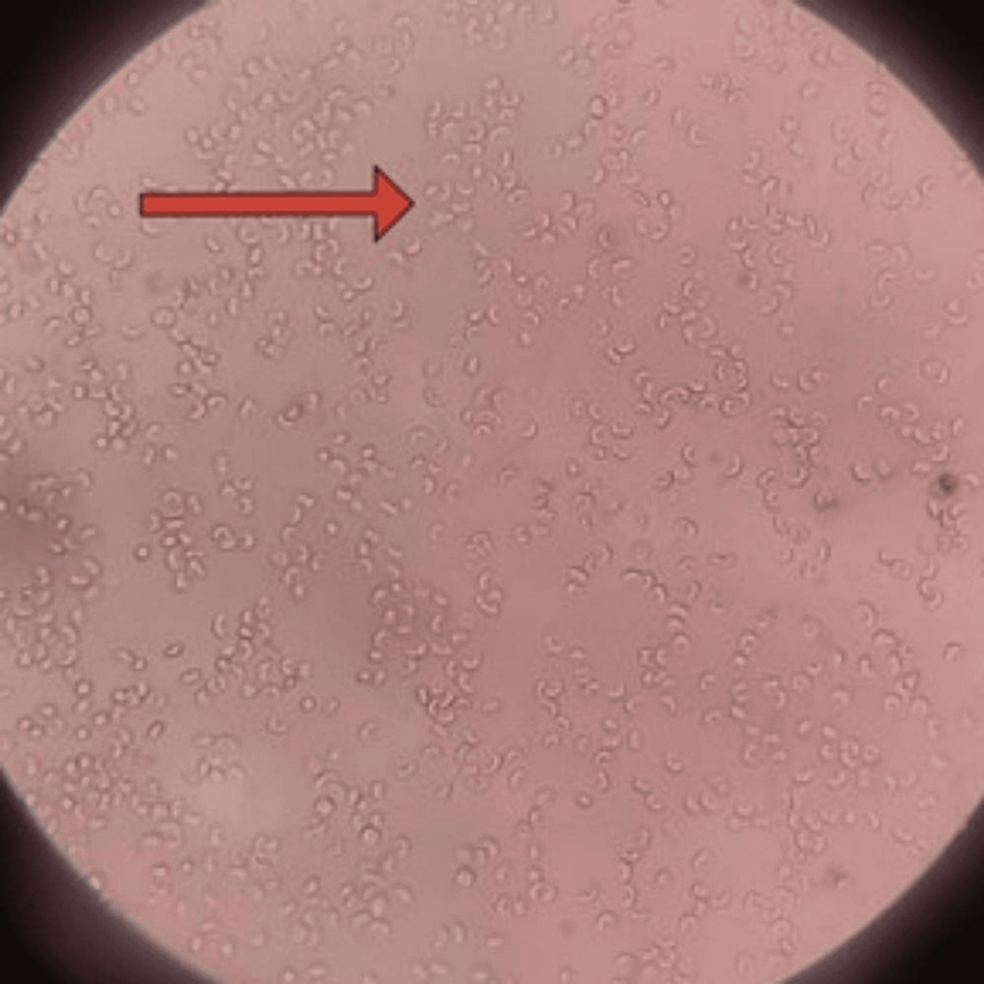
High-power view (40×): Sickling test with 2% sodium metabisulphite (wet preparation) shows sickling of red blood cells (red arrow).

Peripheral smear revealed features of sickle cell anemia (Figure 1). Sickling test revealed sickle cells (Figure 2).

**Figure 3:**
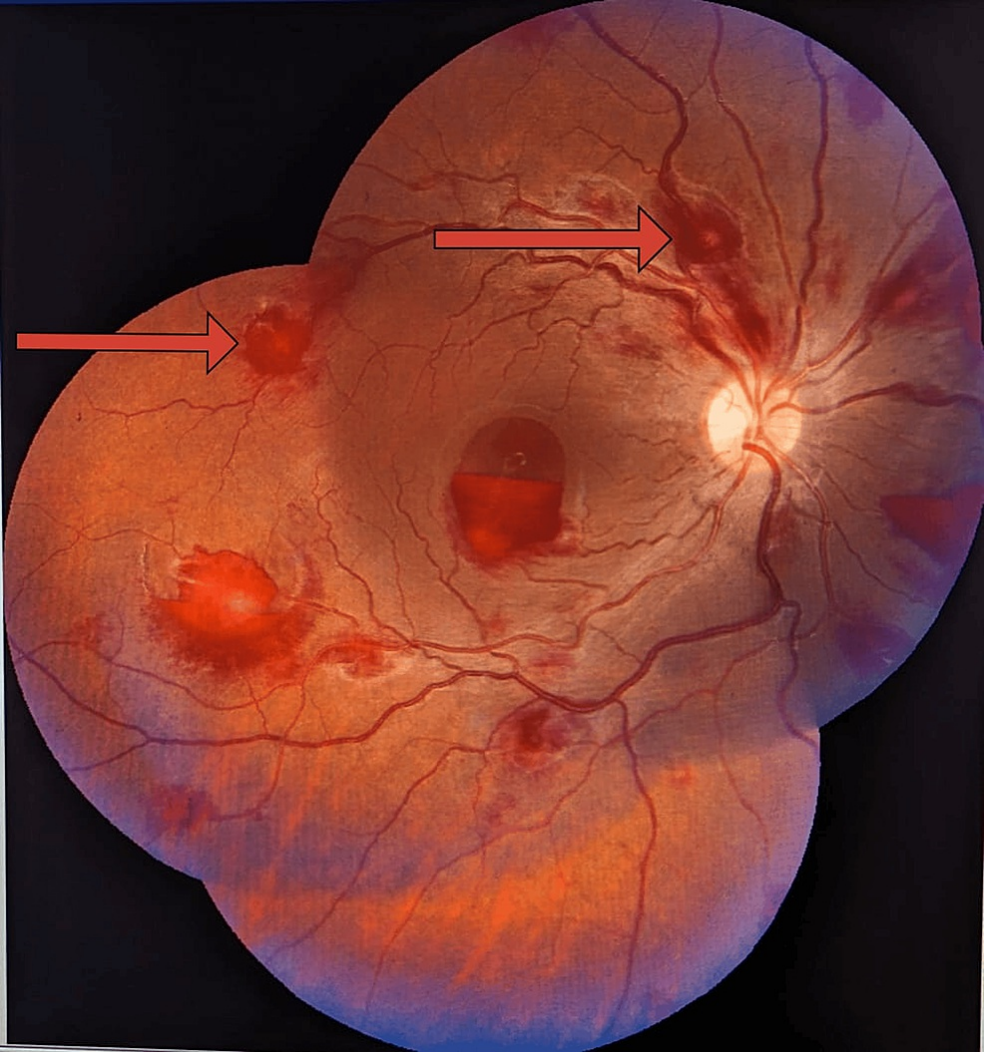
Multiple white-centered retinal hemorrhages (red arrow).

During the hospital stay, the patient complained of a diminution of vision, and a fundus examination revealed multiple white-centered retinal hemorrhages suggestive of Roth spots (Figure 3).

Other causes of Roth spots were ruled out on clinical examination by retrospective evaluation. Blood cultures were sent, which showed no growth in the culture media. Two-dimensional echocardiography was done to look for infective endocarditis, but it was normal.

The patient was started on injectable tramadol and hydration. The patient was transfused with packed red cells until her hemoglobin level increased to 10 g%. Hydroxyurea tablets were given. The patient was asked to follow up in the ophthalmology department after three weeks but was lost to follow-up.

## Discussion

Sickle cell disease has been associated with ocular manifestations, specifically when linked to the retina, they are commonly referred to as sickle cell retinopathy. Retinopathies are the most common complications associated with sickle cell disease.

The pathophysiology behind sickle cell retinopathy involves vaso-occlusion of retinal microvasculature due to the sickled red blood cells, which leads to ischemia and subsequent tissue damage in the retina. Commonly described clinical manifestations of sickle cell retinopathies include peripheral retinal vascular occlusion, retinal hemorrhages, cotton-wool spots, macular Ischemia, and neovascularization.

Our case highlights a rare finding in sickle cell anemia, Roth spots, which are supposed to be the hallmark of infective endocarditis.

Although Roth spots are non-specific red spots on the retina with white or pale centers, they can also occur in several other conditions, such as acquired immunodeficiency syndrome, hypertension, diabetes, collagen vascular disease, and extreme hypoxia. The initial retinal spots discovered in 1872 were thought to result from ruptured nerve fibers [4]. Current investigation reveals that they may consist of infectious agents, localized ischemia, inflammatory infiltration, and coagulated fibrin, including platelets. Usually, a slit-lamp examination, which involves seeing within the eye with an ophthalmoscope, is used to observe them. Our case highlights Roth spots in sickle cell disease, which is a rare phenomenon [5]. Vaso-occlusion and ischemia due to sickling of cells can damage any organ in the body, of which neurological manifestations have crucial importance as they can lead to disability. Hence, neuroimaging plays in role in helping to diagnose and prevent neurological complications.

Effectively managing Roth spots in patients relies on identifying the underlying cause to prevent systemic and ocular complications associated with the primary disease. While small pre-retinal hemorrhages may resolve on their own, larger amounts of blood can lead to macular toxic damage due to prolonged exposure to hemoglobin and iron [6]. Several techniques are available for treating macular subhyaloid hemorrhage, such as vitreal injections of recombinant tissue plasminogen activator, Nd:YAG laser hyaloidotomy, and pars plana vitrectomy. The use of Nd:YAG laser treatment is highlighted for its quick resolution of subhyaloid hemorrhage, potentially avoiding the necessity for more invasive vitreoretinal surgery [7].

## Conclusions

Sickle cell disease is primarily known for its ocular manifestations such as sickle cell retinopathy, which involves retinal vessel occlusion, ischemia, and neovascularization. However, the presence of Roth spots indicates complex ocular manifestations. Roth spots are an unusual finding in sickle cell, and by presenting this unique case, we contribute to the expanding knowledge on the ocular manifestations in sickle cell disease. Early detection and management of Roth spots can help prevent vision-threatening complications and improve overall patient outcomes.
